# Distinct polymicrobial populations in a chronic foot ulcer with implications for diagnostics and anti-infective therapy

**DOI:** 10.1186/1756-0500-7-196

**Published:** 2014-03-29

**Authors:** Can Imirzalioglu, Shneh Sethi, Christian Schneider, Torsten Hain, Trinad Chakraborty, Peter Mayser, Eugen Domann

**Affiliations:** 1 Institute of Medical Microbiology, Justus-Liebig University Giessen, Schubertstrasse 81, D-35392 Giessen, Germany; 2 Department of Radiology, Justus-Liebig-University Giessen, Klinikstrasse 33, D-35392, Giessen, Germany; 3 Department of Dermatology, Venereology, and Allergology, Justus-Liebig-University Giessen, Gaffkystrasse 14, D-35392, Giessen, Germany

**Keywords:** Polymicrobial infection, Foot ulcer, Microbiome, *Gemella morbillorum*

## Abstract

**Background:**

Polymicrobial infections caused by combinations of different bacteria are being detected with an increasing frequency. The evidence of such complex infections is being revealed through the use of novel molecular and culture-independent methods. Considerable progress has been made in the last decade regarding the diagnostic application of such molecular techniques. In particular, 16S rDNA-based sequencing and even metagenomic analyses have been successfully used to study the microbial diversity in ecosystems and human microbiota. Here, we utilized denaturing high-performance liquid chromatography (DHPLC) as a diagnostic tool for identifying different bacterial species in complex clinical samples of a patient with a chronic foot ulcer.

**Case presentation:**

A 45-year-old female suffered from a chronic 5x5cm large plantar ulcer located in the posterior calcaneal area with subcutaneous tissue infection and osteomyelitis. The chronic ulcer developed over a period of 8 years. Culture and DHPLC revealed a distinct and location-dependent polymicrobial infection of the ulcer. The analysis of a superficial biopsy revealed a mixture of *Staphylococcus aureus*, *Proteus vulgaris*, and *Fusobacterium nucleatum*, whereas the tissue-deep biopsy harbored a mixture of four different bacterial species, namely *Gemella morbillorum*, *Porphyromonas asaccharolytica*, *Bacteroides fragilis*, and *Arcanobacterium haemolyticum*.

**Conclusions:**

This clinical case highlights the difficulties in assessing polymicrobial infections where a mixture of fastidious, rapid and slow growing bacteria as well as anaerobes exists as structured communities within the tissue architecture of chronic wound infections. The diagnosis of this multilayered polymicrobial infection led to a microbe-adapted antibiotic therapy, targeting the polymicrobial nature of this infection in addition to a standard local wound treatment. However, a complete wound closure could not be achieved due to the long-lasting extensive destruction of tissue.

## Background

Polymicrobial diseases caused by combinations of different bacteria, as well as viruses, fungi, and parasites are being detected with an increasing frequency [1,2]. There are different ecological features underlying the induction of polymicrobial diseases, such as microbial interference, where (i) one microorganism generates a niche in the host that suppresses the colonization of other microorganisms or synergistic polymicrobial infections, where (ii) one microorganism generates a niche favorable for the infection and colonization of other microorganisms, or where (iii) one microorganism predisposes the host to colonization by other microorganisms as evidenced between some periodontal pathogens [2,3]. Another example of a polymicrobial infection is an additive infection implying that two or more microorganisms can synergistically cause infection as seen in different clinical entities, such as bacteraemia, abdominal abscess, secondary peritonitis [4], soft-tissue infection or fasciitis [5]. Furthermore, many bacteria are able to form biofilms which provide a perfect niche, a bastion, to protect themselves from the host immune system and anti-infective treatment [6].

The evidence of such complex infections is being revealed through the use of novel molecular and culture-independent methods. Indeed, in the clinical case presented in this study, a mixture of fastidious, rapid and slow growing bacteria as well as anaerobes existed as structured communities within the tissue architecture of a chronic wound infection with *G. morbillorum* being the predominant bacterial species. This mixture would probably not have been detected without the use of an additional molecular technology.

## Case presentation

**Figure 1:**
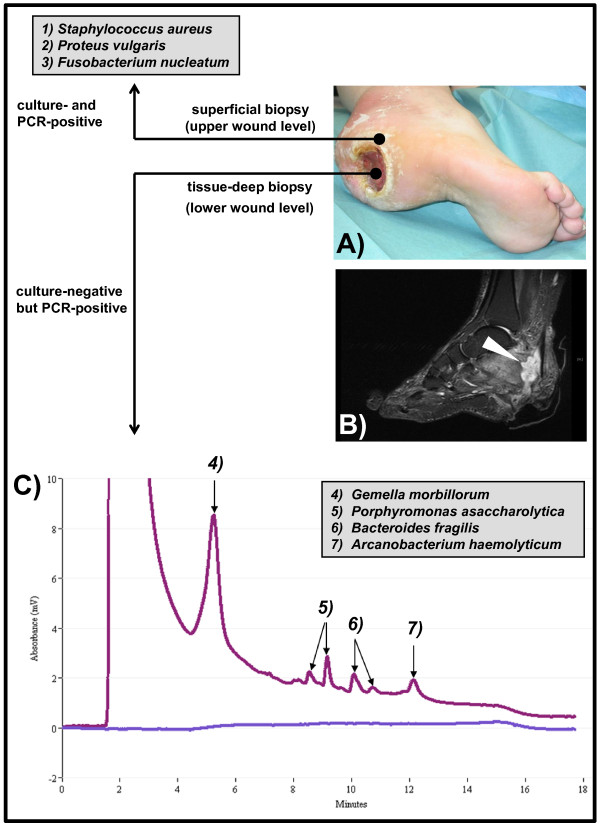
**Distinct polymicrobial populations in the chronic foot ulcer. (A)** Severe plantar ulcer located in the posterior calcaneal area as seen upon consultation. **(B)** Magnetic resonance imaging (MRI) of the left lateral side of the right foot. Multilayered microbial analysis of the ulcer was done by culture, PCR, and denaturing high-performance liquid chromatography (DHPLC). Bacteria (*S. aureus*, *P. vulgaris*, and *F. nucleatum*) were identified from the upper level of the ulcer by a superficial biopsy using both, culture and PCR/DHPLC (top). Bacteria (*G. morbillorum*, *P. asaccharolytica*, *B. fragilis*, and *A. haemolyticum*) were identified from the lower level of the ulcer by PCR and DHPLC analysis only **(C)**.

A 45 year old patient presented herself at the Department of Dermatology, Venereology, and Allergology with a ~5×5 cm large severe plantar ulcer located in the posterior calcaneal area (Figure 1A). She reported acquiring a skin-penetrating injury to her right heel, possibly during a walk along the beach on a holiday trip undertaken in Yucatán/Mexico nine years ago. At that time the injury was treated locally by surgical intervention resulting in a primary wound closure which seemed to early on demonstrate a good healing tendency. However, one year later the patient observed a recrudescence of the wound progressing to an ulceration, which was treated with iodine ointment and gauze bandages solely. With this treatment no significant improvement could be achieved over a course of eight years. Instead, a steady clinical deterioration was evidenced with inflammatory sequelae developing in the surrounding subcutaneous tissue which consequently led to the clinical picture of the wound presented upon consultation at our hospital (Figure 1A).

It was decided to obtain biopsies from superficial sites as well as from deeper tissues for microbiological and histological examination, because of the long lasting medical history and the gradual clinical deterioration of the wound. An intended bone biopsy was declined by the patient. Histological sections were stained with hematoxylin and eosin (HE stain) and a periodic acid-Schiff stain (PAS) was performed. The analysis of the tissue-biopsies from the wound area revealed a granulomatous inflammation and showed signs of chronic infection with PAS being negative. Clinical manifestation of osteomyelitis was suspected due to the deep tissue involvement. Magnetic resonance imaging (MRI) examination revealed inflammatory destruction of calcaneal bone, originating from the posterior, plantar area, thus fitting to the observed clinical picture and confirming the suspected diagnosis of osteomyelitis (Figure 1B). The osteomyelitis was spacious affecting almost the entire tuber calcanei by confluent foci. The Achilles tendon was also affected resulting in a destruction of approximately 50% over a length of 2.5 cm. As similar situations of ulcers and osteomyelitis are frequently observed in chronic diabetic foot infections, an assessment of possible underlying endocrinological causes was carried out. Here, no clinical evidence for diabetes or metabolic syndrome could be found as, among others, HbA1c as a diagnostic marker was unremarkable and blood sugar levels were normal (Table 1). The ulcer also was off odors and obviously impaired her social life. A neurologic evaluation revealed a chronic, idiopathic polyneuropathy, resulting in a sensory impairment also involving pain reception. The suspected reason for the polyneuropathy was a previous herniated disc which also remained untreated. Furthermore, a generalized anxiety disorder was diagnosed which prompted the patient to evade consultations.

**Table 1:** Laboratory tests performed upon consultation

**Tests**	**Values**
**Hematology**	
Leukocytes [giga/l] Eb	**16.8** +
Erythrocytes [tera/l] Eb	5.0
Hemoglobin [g/l] Eb	132
Hematocrit [l/l] Eb	0.40
Thrombocytes [giga/l] Eb	343
MCV [fl] Eb	80
MCH [pg] Eb	**26.6** -
MCHC [g/dl] Eb	33.4
Neutrophils [giga/l] Eb	**11.30** +
Eosinophils [giga/l] Eb	0.30
Basophils [0.03] Eb	0.03
Lymphocytes [giga/l] Eb	3.96
Monocytes [giga/l] Eb	**1.17** +
Erythrocytes sedimentation rate [mm/H] Eb	48/81
HbA1c [%] Eb	6.0
**Clinical chemistry**	
Sodium [mMol/l] Hp	140
Potassium [mMol/l] Hp	4.1
Creatinine [mg/dl] Hp	0.6
Urea [mg/dl] Hp	28
Uric acid [mg/dl] Hp	5.2
Protein [g/l] Hp	81
LDH [U/l] Hp	**256** +
GOT (AST) [U/l] Hp	22
GPT (ALT) [U/l] Hp	20
GGT [U/l] Hp	**41** +
**Plasma proteins**	
CRP [mg/l] Hp	**60.1** +
IgE [IU/ml] Hp	36.1
Antistreptolysin [U/ml] Hp	148

Deviations from the standard values are indicated with a plus (+) sign for increased and a minus (-) sign for decreased values. Both are highlighted in bold type.

For microbiological analysis we obtained both a superficial and a deep subcutaneous tissue biopsy for cultural and molecular examinations (Figure 1A). The samples were routinely processed for culture and for PCR. Briefly, the biopsies were homogenized by using the FastPrep Cell Disrupter (MP Biomedicals, Germany) and firstly streaked on MacConkey, blood, chocolate, Schaedler, and Sabouraud agar plates. The agar plates were incubated at 37°C for two days except for Schaedler and Sabouraud agar plates which were incubated for two weeks and four weeks, respectively. Additionally, an enrichment culture was done in thioglycollate. Secondly, the homogenized samples were subjected to nucleic acid extraction, PCR, and subsequent denaturing high-performance liquid chromatography (DHPLC) as described [7]. DHPLC is a technique proven to be appropriate for the molecular detection of polymicrobial infections. It is capable of separating mixed PCR amplicons derived from a mixture of bacteria in a sample by using a cartridge with microspheres, a fragment collector, amplicon sequencing, and bioinformatics [7-11].

**Figure 2:**
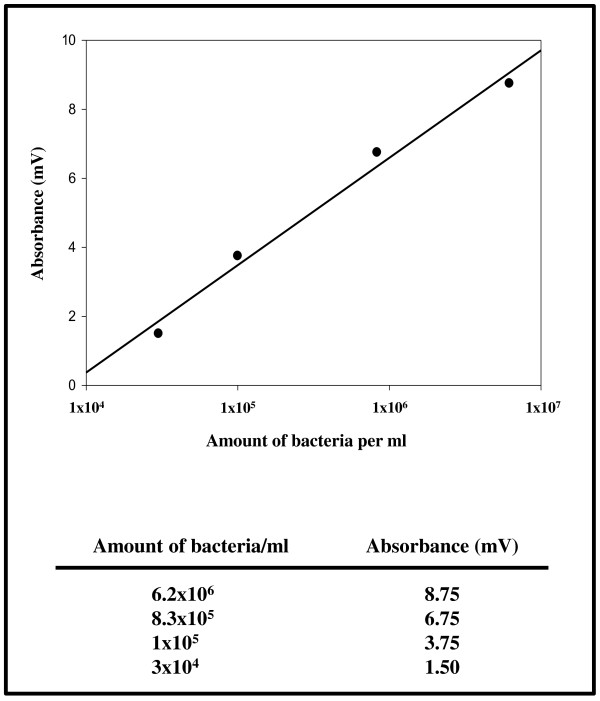
**Standard curve to quantify the amount of bacteria in the biopsies.** The standard curve was generated with *Pseudomonas aeruginosa* as described [7].

Since the patient was in Yucatán/Mexico when she sustained the injury a Bairnsdale or Buruli ulcer caused by *Mycobacterium ulcerans* was suspected. But both microscopy for acid-fast bacteria and a *Mycobacterium*-specific PCR performed from the biopsies were negative. A culture performed on Loewenstein-Jensen at 32°C for 4 months also remained negative. The search for a chronic subcutaneous mycosis, an important differential diagnosis for the patient’s clinical picture, could be excluded by performing fungal culture, dermatohistopathology (PAS, Calcofluor white, Fontana-Masson and Gomori stain), and pan-fungal PCR which all remained negative. However, we did find distinct polymicrobial populations in a superficial and a tissue-deep biopsy which did not overlap. The culture-analysis of the superficial biopsy revealed a mixture of *Staphylococcus aureus*, *Proteus vulgaris*, and *Fusobacterium nucleatum* which was obtained by culture and confirmed by 16S rDNA PCR by using DHPLC. For the tissue-deep biopsy no growth of bacteria using different culture conditions as described above could be obtained. However, these samples were positive for 16S rDNA PCR. Subsequent DHPLC-based analysis permitted the identification of four different bacterial species in the sample, namely *Gemella morbillorum*, *Porphyromonas asaccharolytica*, *Bacteroides fragilis*, and *Arcanobacterium haemolyticum*. DHPLC also enables the quantification of the amount of bacterial rDNA derived from individual species [7]. Therefore, the amount of bacteria per biopsy (~10 mg tissue) was calculated for *S. aureus* as ~10^2^, for *P. vulgaris* as ~10^3^, for *F. nucleatum* as ~10^3^, for *G. morbillorum* as ~5×10^6^, for *P. asaccharolytica* as ~8×10^4^, for *B. fragilis* as ~5×10^4^, and for *A. haemolyticum* as ~5×10^4^ in the samples (Figures 1C and 2).

*G. morbillorum* was identified to be the predominant species in this structured chronic wound infection. It is a gram-positive coccus and a member of the commensal organisms located on the mucous membranes of humans and warm-blooded animals [12]. However, a number of reports have suggested that *Gemella* can cause both severe localized and generalized infections. These infections include abscesses [13,14], endocarditis [15-18], endovascular infections [19,20], infections of the central nervous system [21], septic arthritis [22], and skeletal infections [23]. *Gemella spp*. possesses a typical gram-positive cell wall structure. However, during Gram staining cells easily lose their color and may therefore appear gram-negative (gram-labile). This could probably be the reason for frequent misidentification and the low number of clinical case reports associated with *Gemella* infections. Rapid identification systems based on biochemical features or MALDI-TOF mass spectrometry require cultured bacteria and do not offer reliable identification of all strains of these species once cultivated [24-26].

All of the other bacteria were detected in significantly lower amounts (Figure 1): From the deep-tissue biopsy *P. asaccharolytica*, *B. fragilis*, and *A. haemolyticum* were isolated, which have all been reported to be capable of causing chronic cutaneous or even necrotizing abscesses [27,28]. From the superficial biopsy, which represented the upper level of the wound the clinically well-known and frequently isolated bacteria *S. aureus*, *P. vulgaris*, and *F. nucleatum* were detected. All of these bacteria are known to cause severe wound infections and even necrotizing abscesses [27,29,30].

Interestingly, there were no similarities in bacterial species from either sample suggesting two distinct polymicrobial populations, one located superficially and one located in the deep subcutaneous tissue. Therefore, we postulated that a polymicrobial infection – although not necessarily the primary cause - with predominance of *G. morbillorum* aggravated the lesion, and thereby complicated the healing process. The diagnosis of this multilayered polymicrobial infection prompted a local wound treatment with AQUACELL® Ag (ConvaTec GmbH, Munich, Germany) and Mepilex® Lite (Mölnlycke Health Care GmbH, Vienna, Austria). AQUACELL® Ag is based on an advanced Hydrofiber® technology which locks in fluid and traps bacteria. Incorporated ionic silver exhibits antimicrobial activity and kills a wide spectrum of wound associated bacteria. Mepilex® Lite consists of a thin flexible sheet of absorbent hydrophilic polyurethane foam which is designed for the management of a wide range of exuding wounds such as leg and foot ulcers and which provides a moist wound-healing environment. Furthermore, the diagnosis enabled a microbe-adapted antibiotic therapy, targeting the polymicrobial nature of this infection with emphasis on the predominant pathogen *G. morbillorum* (based on previously reported *Gemella* resistance traits), with clindamycin (daily dose: 3×600 mg) and levofloxacin (daily dose: 1×500 mg) over a period of 6 weeks. Although the ulcer was profuse, deep, and already persisting for nearly a decade the induced treatments prevented further dissemination of the infection, eliminated the potential threat of amputation, and supported tissue regeneration. However, a complete wound closure could not be achieved during the course of this study due to the long-lasting extensive destruction of tissue.

Chronic skin and soft tissue ulcers present challenges regarding diagnosis and treatment. These wounds are frequently caused by a diverse mixture of bacteria encompassing fastidious, rapid and slow growing microbes as well as anaerobes. The traditional detection of such diverse microbes by culture requires dedicated media and culture conditions and is frequently unsatisfactory although the conditions used were appropriate. Therefore, many laboratories also use molecular methods such as PCR to detect pathogens involved in polymicrobial infections. The combination of traditional culture methods and molecular techniques enhances pathogen detection and therefore enables a pathogen-adapted anti-infective therapy. Interestingly, the bacterial community in the superficial biopsy could be cultivated and also detected via PCR. However, the microorganisms in the tissue-deep biopsy were nonculturable. Therefore, the polymicrobial nature of this tissue-deep biopsy was only detectable by culture-independent, molecular methods (PCR and DHPLC). It is known that anaerobes die quickly after sample collection and transport into the laboratory and that diagnosis by culture is therefore difficult [31]. But in our case the transportation times to the laboratory were short and the samples were immediately processed. One would expect that *G. morbillorum* and *A. haemolyticum* were cultivable since the first one is a microaerophilc bacterium, although rather slow growing, and the second one is facultative anaerobe. We hypothesize that the reason for the failure of the culture lies in the potential presence of a polymicrobial biofilm in the deep wound where the microbes existed in a viable but nonculturable (VBNC) status [32,33]. Furthermore, discrepancies between superficial and deep wound samples are often observed in diabetic foot infections, as a model for chronic wound situations, as stated in several publications [34-36]. However, in some of these cases the deep sample or even the bone biopsy remained negative despite clinical evidence of infection. Unfortunately, these samples were not evaluated by molecular methods, as a comparable situation as in this case could be the underlying cause of the culture negativity.

## Conclusions

This clinical case highlights the difficulties in assessing polymicrobial infections where a mixture of fastidious, rapid and slow growing bacteria as well as anaerobes exists as structured communities within the tissue architecture of chronic wound infections. The evidence of such complex infections is being revealed through the use of novel molecular and culture-independent methods. Here, subjecting biopsies obtained from various regions of the wound to DNA-based detection methods provides additional information not available by conventional culture methods. The identification of *G. morbillorum* as a predominant bacterial species associated with the pathogenesis of the wound infection would have probably been missed without the use of an additional molecular technique. Considerable progress has been made in the last decade regarding the diagnostic application of molecular techniques. In particular the use of the 16S rDNA-based sequencing and even metagenomic analyses have been successfully used to study the microbial diversity in ecosystems and human microbiota [37-40]. However, these approaches require sophisticated instrumentation and technologies employing next-generation sequencing machines with subsequent bioinformatic analyses of metagenomic data [41], [42]. At least two hurdles must be overcome for the implementation of these techniques in the clinical diagnostic laboratory. Firstly, these techniques are still time consuming, difficult to reproduce, not yet standardized, and labor-intensive, thereby hindering their routine use in a diagnostic laboratory. Secondly, data linking detection of polymicrobial infection and their utility in assessing clinical states is largely missing [42-44]. Here, we utilized DHPLC as an additional diagnostic tool (staged diagnostics) for identifying different bacterial species in a complex clinical sample. This technology not only allows resolution of mixed amplicons quantitatively, but also permits subsequent sequencing of the individually separated amplicons [7,9]. Falling costs in high-throughput next-generation sequencing technologies and the implementation of enhanced bioinformatic tools, to enable “next-generation diagnostic capabilities” will considerably improve the detection potential of polymicrobial infections in the standard clinical microbiology laboratory.

## Consent

Written informed consent was obtained from the patient for publication of this Case report and any accompanying images. A copy of written consent is available for review by the Editor of this journal. The study was approved by the Ethics Board of the Justus-Liebig-University of Giessen.

## Competing interests

The authors declare that they have no competing interests.

## Authors’ contributions

CI and ED did the microbial examinations, drafted and wrote the manuscript and generated the figures. PM took care of the patient, supervised the clinical guidance and prompted along with CS the medical examinations. Both contributed to the conception of the manuscript and the discussion. SS and TC participated in the conception and writing of the manuscript. TH supervised the sequencing and bioinformatics of the PCR products and contributed to the discussion. All authors read and approved the final version of the manuscript.
